# Politics and Prejudice: How Political Discussion With Peers Is Related to Attitudes About Immigrants During Adolescence

**DOI:** 10.3389/fsoc.2019.00070

**Published:** 2019-10-04

**Authors:** Andrea Bohman, Mikael Hjerm, Maureen A. Eger

**Affiliations:** Department of Sociology, Umeå University, Umeå, Sweden

**Keywords:** prejudice, longitudinal, anti-immigrant, adolescent, discussion, political

## Abstract

Research on prejudice has shown that with whom we surround ourselves matters for intergroup attitudes, but these studies have paid little attention to the content of those interactions. Studies on political socialization and deliberation have focused on the content of interaction by examining the transmission of norms as well as the direct consequences of political discussion on attitudes and behavior. However, this literature has not focused on prejudice as a potential consequence. In this study, we combine these approaches to examine if political discussions with peers during adolescence matter for prejudice. We rely on five waves of a Swedish panel of adolescents, ages 13–22. Results show an association between political discussion and prejudice over time, and that this relationship increases as adolescents grow older. Results also demonstrate that the effect of political discussions depends on the level of prejudice in one's peer network. Discussion with low prejudice friends is associated with lower levels of prejudice over time, while political discussion with high prejudice peers is not significantly related to attitudes.

## Introduction

Often described as the “impressionable years” (Krosnick and Alwin, 1989), adolescence is the time when many social and political attitudes develop (Alwin and Krosnick, 1991; Rekker et al., 2015), including ethnic and racial prejudice (Coenders et al., 2008). Importantly, these attitudes are not formed and maintained in isolation. With whom we surround ourselves influences how attitudes develop, something that is especially true during the formative adolescence years (Berndt, 1979). In adolescence, attitudes become increasingly susceptible to social influences, as evidenced by a recent meta-study of age trends in ethnic and racial prejudice (Raabe and Beelmann, 2011). Findings suggest that while biological processes are important drivers of attitudinal development in young children, social factors are central to prejudicial attitudes among adolescents. In addition, twin studies (Hatemi et al., 2009; Orey and Park, 2012) show that although genetic influence play a role in adolescence, environmental factors are more important for the development of attitudes. Indeed, Orey and Park (2012) conclude that unique environments explain 82% of the variation in ethnocentrism leaving only 18% to inheritance. These studies lend credence to the notion that social influence is paramount for the development of prejudice.

A large body of research demonstrates that significant others such as parents and peers play an important part in this process (e.g., Sinclair et al., 2005; Hogg and Smith, 2007). Social influence occurs as adolescents observe and interact with key figures in their immediate surrounding, some of which may be more “significant” than others. Indeed, studies show that adolescents' attitudes are particularly susceptible to peer influence (Berndt, 1979; Thijs et al., 2016), a relationship consistently observed in research on prejudicial attitudes (e.g., Poteat et al., 2007). Not only do friends tend to display similar levels of prejudice (Kiesner et al., 2003), socializing with prejudiced peers also increases negative out-group attitudes over the course of adolescence (Hjerm et al., 2018).

While we know from previous empirical research that individual attitudes are susceptible to social influence, especially during adolescence, there are still important gaps in our understanding of how such influences occur. In particular, we know little about *how* the ways we interact with other people influence prejudice, despite major theories' focus on the social context. According to social learning theory, attitudes are learned from observing other peoples' actions and the consequences of these actions (e.g., Bandura, 1977). Arguably, interpersonal interactions and communication are implicit in the account, however studies have not tested this empirically. The same can be said of studies based on intergroup contact theory (e.g., Pettigrew, 1998). According to this theory, contact with members of an out-group facilitates positive attitudes toward the out-group in question. The quality of intergroup contact is an important feature of the theory, but this is typically operationalized as the circumstances under which people have contact (e.g., friendship, acquaintanceship, or professional relationship) and not necessarily what the social interaction actually entails. Put differently, previous empirical research on social influence and prejudice has primarily focused on the impact of with *whom* we surround ourselves, either in terms of their ethnic and racial background (Pettigrew and Tropp, 2011; Hooghe et al., 2013) or in terms of their intergroup attitudes (Poteat et al., 2007; Mitchell, 2019). Instead, this article focuses explicitly on one form of social interaction: discussion. Specifically, we are interested in how political discussion among peers influences the development of prejudice during adolescence.

Despite the fact that neither focuses explicitly on prejudice, both the literatures on political socialization (McLeod, 2000; McDevitt, 2006) and on deliberative democracy (Fearon, 1998) recognize political discussion as important for attitudes and behavior. Therefore, we make use of these literatures to identify two reasons why political discussions could affect prejudice among adolescents. First, the act of discussion itself may engender the development of moral and civic values, making prejudice less likely; and, second, adolescents may be influenced by the content of discussion, which is partly determined by the attitudes of their significant others. The current study tests both of these hypotheses. Additionally, we also assess at which age, during the impressionable years, those discussions matter most.

To determine if political discussions influence the development of prejudicial attitudes during adolescence, we rely on a panel of Swedish adolescents aged 13–22. Collected annually for five waves, this longitudinal dataset contains questions about social interaction and communication and also includes the attitudes of respondents' parents and peers. With these data, we investigate: (1) the relationship between political discussions and anti-immigrant attitudes; (2) how the size of the association between political discussions and anti-immigrant attitudes changes with age; and (3) the interaction between political discussion and peers' attitudes in the development of anti-immigrant attitudes over time.

## Political Discussion and Prejudice

Early theorizing on social influences suggests that interpersonal discussions can play an important part in the development of social and political attitudes (Deutsch and Gerard, 1955). Previous work on political discussions suggests two main ways that this type of interpersonal interaction can affect prejudice in adolescence. First, discussion may function as deliberation and second, discussion may function as a transmitter of attitudes from peers to the adolescent. While the latter implies an interaction between attitudes and discussions, the former suggests the possibility that the very act of discussing politics may have implications for the development of individual attitudes.

According to the literature on deliberative democracy (Bessette, 1980), democracy at its essence is deliberation, as opposed to voting or constitutional rights. By this account, any form of communication that induces reflection and that is not coercive is deliberation (Dryzek, 2000). To deliberate, or to “weigh the merits of competing arguments in discussions together” (Fishkin, 2011, p. 33), stimulates the participants' moral and intellectual qualities. Its interactive nature provides opportunities to consider issues from other peoples' vantage point, facilitating the development of emphatic concern and perspective-taking abilities (Fearon, 1998; Price et al., 2002). In this sense, discussions hold the potential to expand individuals' knowledge about the world and to contribute to the development of important civic and human values, which may also have consequences for the development of prejudice or its opposite.

Indeed, empirical research demonstrates that as people become better equipped to imagine how they would think and feel from another person's perspective, they also become less likely to hold prejudicial attitudes (Galinsky and Moskowitz, 2000; Nesdale et al., 2005; Butrus and Witenberg, 2013; Miklikowska, 2018). In line with this, studies have found a positive relationship between participation in political discussions and tolerant attitudes amongst adults. Studying political discussions at the workplace, Mutz and Mondak (2006) demonstrate a positive relationship between the frequency of political discussions and political tolerance. Being frequently exposed to different types of arguments both increased the workers' knowledge about and fostered appreciation for the rights of groups with which they personally disagreed. Similarly, Pattie and Johnston (2008) found that adults who often participate in political discussions are more likely to tolerate political views and lifestyles that are different from their own. Broockman and Kalla (2016) show that conversations that encourage perspective taking with regard to an outgroup can have a lasting effect on prejudice. Based on this research, we first test the hypothesis that:

***H1:*** *The frequency of engaging in political discussions with friends is inversely related to anti-immigrant attitudes during adolescence*.

## When Does Political Discussion Matter?

Although parents matter for adolescents' levels of prejudice, parents' social influence decreases over the course of the formative years (e.g., McLeod and Shah, 2009). This is partly because adolescents tend to spend less time with their parents as they grow older (Larson et al., 1996) and partly because they confide less in their parents (Keijsers et al., 2009). Quintelier (2015) shows that peers are more important than parents and school in terms of political participation in late adolescence. Similarly, Gotlieb et al. (2015) demonstrate that, in late adolescence, the direct influence of socializing agents and background characteristics on political behavior diminishes compared to the effect of communication with peers.

Not only does the impact of socialization change with increasing age, but so do adolescents' capacity for more nuanced discussions and ability to absorb such discussions. In the 1960s, Adelson and O'Neil (1966) interviewed adolescents in various age groups and concluded that older adolescents are more susceptible to more complex political discussions where political judgments are based on philosophical ideas. Moreover, older children are in general more affected by communication than younger children in terms of political socialization (Eveland et al., 1998), which likely is due to increasing cognitive maturation (e.g., Luna et al., 2004). Thus, political discussion with peers becomes increasingly important for two reasons. First, the relative importance of peers as agents of influence increases over time, and second, the capacity of adolescents to engage in and absorb nuanced discussion increases with age. We contend that political discussions should have similar consequences for prejudice. Thus, we hypothesize:

***H2****: The relationship between political discussions with friends and anti-immigrant attitudes increases with age*.

## Political Discussion And Peer Prejudice

While our first two hypotheses posit how and when political discussions can reduce negative outgroup attitudes, we also have strong reasons to expect this effect to be dependent on norms or attitudes of the discussants. The social aspect of attitude formation implies that individuals tend to adjust their views and perceptions to attitudes held by people in their immediate surroundings (Bandura, 1977; Crandall et al., 2002). Discussions, in this context, become important primarily as a forum for the transmission of attitudes. This is a central theme in political socialization research, which consistently have demonstrated that communication and discussion are critical for the transmission of norms and values (e.g., McLeod, 2000, McDevitt, 2006). Political socialization is the process by which individuals become civic-oriented participants in liberal democracy, and studies show that communication is an important part of this process, including communication via mass-media (Sears and Valentino, 1997), within families (Niemi and Jennings, 1991), between peers (Quintelier, 2015) and within schools (Castillo et al., 2015).

As for political discussion specifically, studies on parent-child similarity find that the intergenerational transmission of attitudes strongly depend on the degree of political discussion in the family (Meeusen, 2014; Hooghe and Boonen, 2015). In families that frequently discuss social and political issues, children generally resemble their parents more than in families where political discussions are rare (Jennings et al., 2009). This relationship also applies to the transmission of prejudicial attitudes (Meeusen and Dhont, 2015). Experimental studies suggest that schoolchildren can become less prejudiced after being faced with alternative perspectives via discussion with others (Aboud and Doyle, 1996; Aboud and Fenwick, 1999). Further, the literature on group polarization demonstrates that discussion with others may push ingoing attitudes toward extreme positions (Myers and Lamm, 1976; Isenberg, 1986; Binder et al., 2009). Group discussion tends to exaggerate the discussants' preferences, so that the average post-discussion position of the group is more extreme, in that it deviates more from neutrality than the average pre-discussion position (Moscovici and Zavalloni, 1969). The shift occurs in the direction of the initial attitudes, which in the context of anti-immigrant attitudes suggests that groups that initially feel some hesitation toward immigrants, via discussion, will develop even more negative attitudes (and vice versa) (Myers and Bishop, 1970).

Despite this scholarship, there is no unified theoretical framework to explain transmission of attitudes via political discussion. Yet, other scholarship provides guidance in understanding how groups exert influence via discussion. In an early account, Deutsch and Gerard (1955), identify two modes of influences which have been formative to the literature on social influences (Turner, 1991). Normative social influence, first, occurs as people align with other's preferences to gain social rewards and avoid social sanctions (Deutsch and Gerard, 1955; Kaplan and Miller, 1987). The desires to be accepted and liked by the group and simultaneously avoid sanctioning, drive the tendency to conform to other group members' expectations. Informational influence, on the other hand, occurs as group members compare their views and adjust their preferences based on a desire to be correct (Asch, 1956; Price et al., 2006). Information provided by other members is read as evidence about reality (Deutsch and Gerard, 1955) and attitudes shift in response to arguments put forward by group members (Burnstein and Vinokur, 1977).

While both informational and normative accounts attribute attitudinal shifts primarily to external constraints (sanctions/rewards + argument quality), a third approach emphasizes the role of internalized group norms associated with valued social identities. According to work that draws on social identity theory (Tajfel and Turner, 1979) and group norm theory (Sherif and Sherif, 1953), individuals align their attitudes and behavior with that of their friends to connect socially with the group (Crandall et al., 2002; Hogg and Smith, 2007). This occurs via referent informational influence (Turner, 1981; Abrams and Hogg, 1990) a process where people confirm their in-group membership by internalizing the perceived group norm associated with specific social identities. The process unfolds in three steps: (1) people categorize themselves as belonging to distinctive social group (-s); (2) they form an understanding of the in-group norm; and (3) enact their understood role as group members by conforming to this norm (Abrams and Hogg, 1990). Discussions primarily contribute to the second step, as the content of valued social identities and group norms takes shape in intragroup and intergroup interactions. Thus, although referent informational influence primarily is an internal process, people must have an understanding of the norm. Hogg and Smith (2007, p. 98) explain: “Although people have a general idea of what is normative, they look to others for confirmation of what is situationally normative—they use the behavior and expressed attitudes of others to determine situationally relevant ingroup normative attitudes (p. 98).” In sum, these different accounts direct attention to the content of discussions. They suggest that what is being said, due to the attitudes of the in-group/fellow discussants, will influence how (i.e., in what direction) the attitudes develop, while the degree of discussions will impact to what extent it occurs.

Thus, we test a third and final hypothesis:

***H3****: The relationship between political discussions with friends and anti-immigrant sentiment depends on friends' level of prejudice*.

## Data and Method

We use data from the Youth and Society dataset (Amnå et al., 2010), a Swedish longitudinal panel that consists of five cohorts. Given our focus on the formative years, we rely on a sub-sample of the data covering only the two youngest cohorts, aged 13 (*M* = 13.41, *SD* = 0.54) and 16 (*M* = 16.56, *SD* = 0.62) at time 1 (T1). The initial sampling was based on schools (13 junior high schools and high schools), selected to be socially and ethnically representative. Cohort 1 respondents were surveyed on a yearly basis for all 5 years, 3 years while in compulsory junior high school and 2 years while in non-compulsory high school. Respondents in cohort 2 were surveyed four times over the 5-year period, 3 years while in high school and 1 year after they had left school. Cohort 2 respondents did not participate at time 4 (T4). Data collection occurred between 2010 and 2014 in a mid-sized Swedish city, where the unemployment rate, average income level, and the relative size of the immigrant population are comparable to national averages.

Response rates at T1 were 94% in cohort 1 (*n* = 904) and 85% in cohort 2 (*n* = 892). Attrition rates are not trivial (23% over five waves for cohort 1 and 52% over four waves for cohort 2), but comparable to other longitudinal studies on adolescents (Stearns et al., 2009; Dejaeghere et al., 2012). The largest drop in participation for cohort 2 occurs between T3 and T5 (38%), which coincides with its graduation from high school. Importantly, attrition is not significantly related to any variables of interest. Mean scores in prejudice at T1 for respondents who participated at T5 are no different from mean scores at T1 for those that did not participate at T5 (*M* = 2.20, SE = 0.023; *M* = 2.26, SE = 0.028). Moreover, we run all models in the analysis only on respondents present at T5 (*n* = 850). These analyses, available upon request, confirm the findings from the full sample.

### Dependent Variable

We operationalize prejudice by measuring adolescents' attitudes toward immigrants. While prejudice is a broader construct that can mean negative attitudes toward a variety of out-groups (based on gender, race, age, sexual orientation, disability, religion, or nativity), we focus specifically on anti-immigrant attitudes. In the European context, immigration is highly salient as it is the main engine of increasing diversity on the continent. Further, “immigrants” are the most common out-group in European studies of prejudice, a literature to which we aim to contribute. We measure anti-immigrant attitudes using an index based on three items in the *Youth and Society* dataset. These are: “Immigrants often come here just to take advantage of welfare in Sweden”; “Immigrants often take jobs from people who are born in Sweden”; and “It happens too often that immigrants have customs and traditions that not fit into Swedish society.” Similar items are available in European Social Survey (ESS 2002-2016) and have been used to measure anti-immigrant attitudes in past research (e.g., Schneider, 2008; Hjerm, 2009). At each wave, respondents reported to what extent each of the three statements corresponds to their own position by marking their answer on four-point scales, ranging from 1 indicating “Doesn't apply at all” to 4 indicating “Applies very well.” We use row means to generate a dependent variable that varies between 1 and 4, with higher scores indicating higher levels of anti-immigrant attitudes. Over the five waves, the Cronbach's alpha varies between 0.75 and 0.81, indicating internal reliability. Also, previous research that uses these data tested for metric invariance, noting that the items capture the same underlying phenomena across waves (Hjerm et al., 2018). See Table A1 for descriptive statistics.

### Independent Variable

We use two items to capture political discussion. Both begin with the question: “How often does it happen that you and your friends talk about the following things?” and capture the extent to which respondent discuss (1) “what you have heard on the news about what is going on in Sweden and around the world” and (2) politics or societal issues. Four responses were available for both questions, ranging from 1 indicating “Very often” to 4 indicating “Never.” We reversed the scale and combined the two items using the mean item score. Thus, the measure of political discussions varies between 1 and 4, with higher scores indicating more political discussions.

### Moderators and Main Controls

To test hypothesis 2 and 3, we require information about the respondents' age and level of prejudice among their peers. The respondents' age is provided in the dataset, but to facilitate interpretation of the results we center the age variable on its grand mean. This step ensures that “zero” corresponds to an actual observed value (now the sample's average age). Friends' attitudes are facilitated by peer nominations. At each wave, adolescents were asked to identify up to eight best friends. 94% of the adolescents nominated at least one friend at T1 and 74% at T5. In most cases, adolescents nominated friends who were already part of the sample. If nominated friends were not part of the original study, they were snowballed into the sample and asked the same questions as the original target group. Response rates in the snowball sample were 57% (*n* = 249) at T1 and 68% (*n* = 967) at T5. Friends' prejudice is captured by the same measure as the dependent variable. Based on the friends' prejudice scores, we calculate the average level of “anti-immigrant attitudes” among nominated friends for each respondent, at each wave, producing a time-variant independent variable. As our main independent variable asks about discussions with friends in general and our measure of friends' prejudice is based on nominated friends, there may be some discrepancies in whom respondents think of when answering the questions. Still, we have no theoretical or practical reason to assume that the adolescents have different friends in mind.

Besides age and friends' prejudice, we also control for own interest in politics. We use two questions in the dataset to generate an index: “How interested are you in politics?” and “How interested are you in what is going on in society?” The scale for both item ranges between 1 indicating “very interested” and 5 indicating “totally uninterested.” Before averaging the item scores we reverse the scale so that higher numbers denote more interest. We also run our models with a set of additional controls, including gender, time-variant indicators of social isolation, other discussions with peers, political discussions with parents as well as indicators of parents' prejudice and educational level. We present these in more detail in Table A1.

### Analytical Approach

To test our hypotheses about political discussion on anti-immigrant attitudes, we analyze data with mixed, multilevel repeated measurement models. These are hierarchical models, with time nested in individuals. This approach considers different starting values and different trajectories over time, thereby controlling for previous time points and, more importantly, starting points. The generic model looks like this:

$$\begin{array}{l} Y_{ti}=\beta +\beta X_{i}+X_{ti}\;\left(\beta +u_{i1}\right)+u_{io}+e_{ti} \end{array}$$

*Y*_*ti*_ is the *t*th response for *i*th subject. The β′s are the beta-coefficients, including an intercept. *X*_*i*_ is a time invariant variable and *X*_*ti*_ is a time variant variable. The *u*′s are the random effects for each *i*, *u*_0_ being the random intercept and *u*_1_ a random slope. *e*_*ti*_ is the residual variance at level 1.

We specify a first order autoregressive covariance structure for the within-individual part of the model. This means that we expect that two adjacent time points are more highly correlated than two non-adjacent time points, but that the correlation between T1 and T2 is the same as between T4 and T5. This error structure generates the best model fit^1^.

It is important to know whether within-individual change or between-individual differences are responsible for the relationships between our dependent variable and key independent variables. To do this, we create two new orthogonal variables from each independent variable.

To capture between-individual effects for independent variables, we use all rounds to create an average for each person. Then, we subtract this variable from the sample grand mean. The resulting variable is the difference between the respondent's average value over all rounds and all respondents' average value. To capture within-individual effects for independent variables, we subtract a respondent's raw score for each time point from the respondent's mean score across all time points. Included together in the analysis, these two variables enable us to separate between-individual effects from within-individual effects. Without this transformation, coefficients would merely represent the average effect of within-individual and between-individual differences. Therefore, we do this for all time-variant covariates except age.

## Results

**Figure 1:**
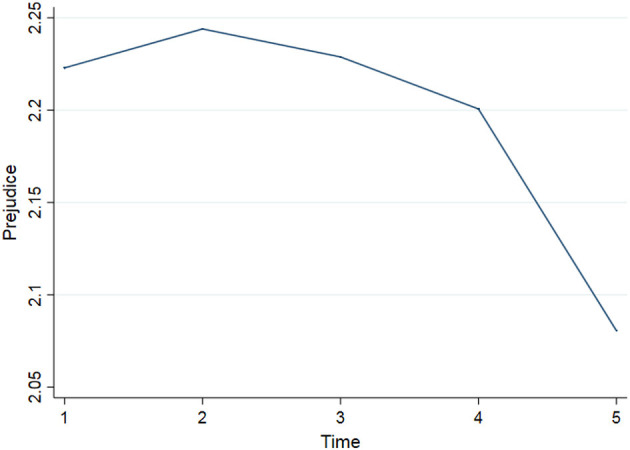
Average levels of prejudice T1-T5.

**Figure 2:**
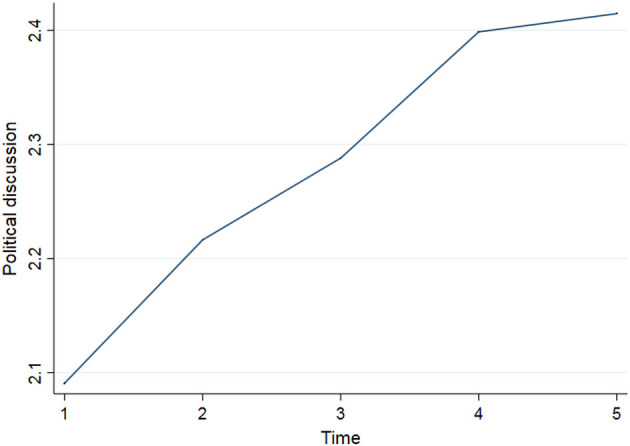
Average levels of political discussion T1-T5.

As illustrated in Figure 1, the average trend in anti-immigrant attitudes is curvilinear in shape. Attitudes toward immigrants are most negative at T2 and T3 and most positive at T5. Figure 2 shows that our main independent variable, political discussion with peers, increases almost linearly over time as respondents get older^2^.

Table 1 reports results from repeated measurement models. Model 0 displays the effect of time as dummies. The random part of the model reveals between-person differences both in initial levels of anti-immigrant attitudes and in the rate of change. There is significant variation around the effect of time, which suggests adolescents differ in how their attitudes develop over the five waves. Rho tells us that the correlation between any two adjacent time-points is 0.27, i.e., when including random slope and intercept.

**Table 1:** Political discussions and prejudice, linear mixed models with repeated measurements.

	**Model 0**	**Model 1**	**Model 2**	**Model 3**	**Model 4**
**Fixed**					
Intercept	2.30 (0.02)^***^	2.29 (0.02)^***^	2.22 (0.06)^***^	0.86 (0.10)^***^	2.25 (0.04)^***^
T1 (ref)					
T2	0.02 (0.02)	0.03 (0.02)	0.10 (0.06)	0.06 (0.06)	0.10 (0.06)
T3	0.02 (0.02)	0.03 (0.02)	0.08 (0.02)	0.03 (0.06)	0.08 (0.06)
T4	0.03 (0.03)	0.04 (0.03)	0.10 (0.03)	0.05 (0.07)	0.10 (0.06)
T5	−0.14 (0.03)^***^	−0.12 (0.03)^***^	−0.09 (0.07)	−0.14 (0.07)^*^	−0.10 (0.07)
Political discussion (w)		−0.05 (0.02)^**^	−0.05 (0.02)^**^	−0.05 (0.02)^**^	−0.05 (0.02)^**^
Political discussion (b)		−0.26 (0.03)^***^	−0.14 (0.04)^***^	−0.15 (0.04)^***^	−0.13 (0.04)^***^
**Controls**					
Friends' prejudice (w)			0.20 (0.03)^***^	0.20 (0.03)^***^	0.20 (0.03)^***^
Friends' prejudice (b)			0.63 (0.04)^***^	0.63 (0.04)^***^	0.62 (0.04)^***^
Political interest (w)			−0.02 (0.01)^*^	−0.01 (0.01)	−0.02 (0.01)^*^
Political interest (b)			−0.07 (0.02)^**^	−0.06 (0.02)^**^	−0.07 (0.02)^**^
Age			0.03 (0.01)^**^	0.03 (0.01)^**^	0.03 (0.01)^***^
**Interactions**					
Age* Political discussion (b)				−0.04 (0.01)^***^	
Friends' prejudice (b) ^*^Political discussion (b)					0.25 (0.06)^***^
**Random**					
Time	0.11 (0.01)	0.09 (0.01)	0.05 (0.02)	0.05 (0.02)	0.05 (0.02)
Intercept	0.47 (0.02)	0.44 (0.02)	0.34 (0.03)	0.34 (0.03)	0.33 (0.03)
**Residual (Ar1)**					
Rho	0.27 (0.04)	0.30 (0.04)	0.38 (0.04)	0.39 (0.04)	0.38 (0.04)
Sd (e)	0.53 (0.01)	0.54 (0.02)	0.57 (0.02)	0.57 (0.02)	0.57 (0.02)
*n*	1,481	1,480	1,442	1,442	1,442
obs	4,974	4,966	4,378	4,378	4,378
BIC	9554.526	9460.308	8030.581	8027.893	8024.332
AIC	9495.919	9388.694	7928.431	7919.359	7915.798

*Standard errors in brackets*.

* p < 0.05;

** p < 0.01;

*** *p < 0.001. (w), within-person effects; (b), between-person effects*.

Model 1 tests our first hypothesis that the frequency of engaging in political discussions with friends is inversely related to anti-immigrant attitudes. Based on literature linking political discussions to the development of civic and moral virtues, including individuals' perspective-taking ability (Fearon, 1998), we expect adolescents who frequently discuss politics with their friends to be less likely to hold anti-immigrant attitudes. Relatedly, we expect a relationship between within-person changes in the frequency of political discussion and within-person changes in anti-immigrant attitudes. Results largely support H1. There is a significant negative between-person effect of political discussions on prejudice (*b* = −0.26 SE = 0.03). A one-unit increase in political discussions corresponds to quarter of a unit decrease in anti-immigrant attitudes, indicating that those who frequently engage in political discussion with friends are less prejudiced than adolescents who do not. Within-person changes in discussion also make adolescents slightly less prone to hold anti-immigrant attitudes (*b* = −0.05 SE = 0.02)^3^. However, this coefficient is small, suggesting that discussion primarily explains between-adolescent differences in prejudice.

In model 2, we add controls, which does not change our findings. Variation in friends' prejudice, own political interest, and age partly account for the between-person effect of discussion; nevertheless, the hypothesized relationship remains robust. Friends' prejudice relates to the level and the development of anti-immigrant attitudes in expected ways. Adolescents who socialize with friends who are high in prejudice are also more likely to express such attitudes (*b* = 0.63 SE = 0.04) and fluctuations in friends' attitudes predict within-person changes in anti-immigrant attitudes (*b* = 0.20 SE = 0.03). Both of these results are consistent with previous research (Hjerm et al., 2018; Miklikowska et al., 2019). Thus, friends are important agents of social influence in regards to both the level of prejudice and how these attitudes develop over time. One's own political interest is negatively related both to between-person differences (*b* = −0.07 SE = 0.01) and to within-person changes in anti-immigrant attitudes (*b* = −0.02 SE = 0.01). The positive effect of age implies that when we account for the other controls, including the general development over time, adolescents become slightly more prejudiced as they grow older.

In summary, results from models 1 and 2 largely support H1. In robustness checks, we control for additional covariates (see appendix Table A2 for full model). These models show that the within-person effect of political discussions cannot be separated either from the effect of (1) within-person fluctuations in peer discussions on other topics or from (2) within-person fluctuations in political discussions with parents. Although these controls emerge as unrelated to anti-immigrant attitudes, when modeled together with own political interest, they still cancel out the significant effect of within-person changes in political discussions. It is debatable whether it is reasonable to expect an effect of political discussion beyond fluctuations in these closely interrelated features. Still, future research should attempt to disentangle their independent effects and/or determine how they may work in concert to influence prejudice in adolescence. Importantly, the between-person effect of political discussions is stable in all models, including when controlling for different measures of social isolation (popularity in terms of friendship nominations, number of reciprocated friendship nominations^4^, and feeling of loneliness in the class), discussions with friends on other topics (movies, weekend activities, school, the environment, social media and games), political discussions with parents, parents' education as well as parents' attitudes toward immigrants.

**Figure 3:**
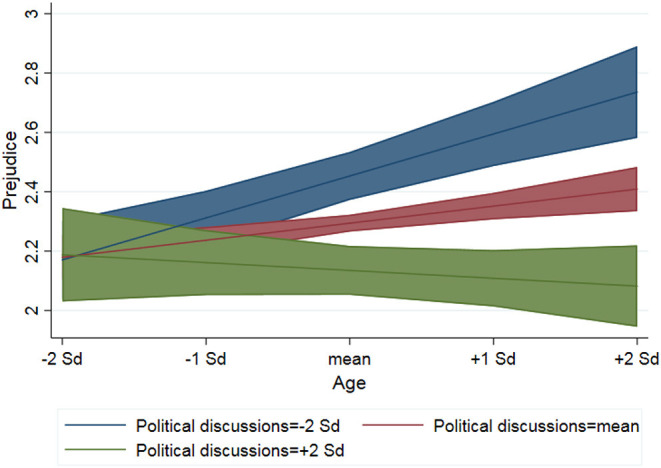
Predicted values from linear mixed repeated measurement model (model 3) with 95% confidence intervals.

To test hypothesis 2, we include an interaction term between age and political discussions in model 3. Results demonstrate that as adolescents grow older, the negative effect of political discussions become stronger. Thus, consistent with the theoretical expectations based on growing significance of peers and increasingly complex discussions, we find that the older the adolescents get, the more effectively do political discussions with friends reduce anti-immigrant attitudes^5^. Figure 3 illustrates this relationship, revealing that for the youngest in the sample (individuals in cohort 1 at T1) there is no statistically significant difference in prejudice between those who engage in political discussion and those who do not. Indeed, political discussions become more consequential for anti-immigrant attitudes as adolescents grow older.

**Figure 4:**
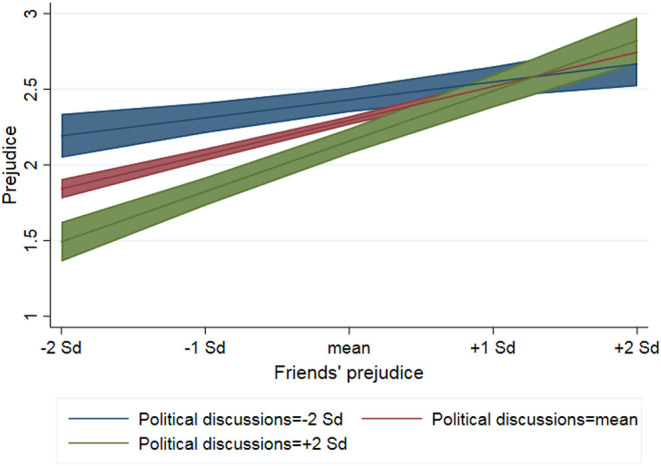
Predicted values from linear mixed repeated measurement model (model 4) with 95% confidence intervals.

Model 4 examines the role of political discussions as social influence among in-group members. Results provide support for hypothesis 3, which expects the effect of political discussions on prejudice to depend on friends' attitudes. While previous models have demonstrated that a high degree of political discussions is associated with less anti-immigrant attitudes, the interaction in model 4 shows that this becomes less true in high prejudice peer groups (*b* = 0.25, SE = 0.06). In fact, as shown in Figure 4, the negative effect of political discussions is most visible among adolescents whose friends have <1 standard deviation above the average degree of anti-immigrant attitudes. In line with observations in experimental studies (Aboud and Doyle, 1996; Aboud and Fenwick, 1999), prejudicial attitudes appear to be influenced primarily by discussions with low-prejudice friends. That political discussions are unrelated to anti-immigrant attitudes if friends are very high in prejudice cannot be explained by greater attitudinal homogeneity among high prejudice peers, as the dispersion of attitudes is not significantly lower in these groups.

## Conclusion

Previous research tells us *which* significant others matter for the development of attitudes during adolescence (e.g., Paluck, 2011) and that peer prejudice is associated with individual prejudice over time (e.g., Poteat, 2007; Hjerm et al., 2018). With this research, we aim to examine how a particular kind of social interaction is related to the development of prejudice over time. More specifically, we examine the consequences of engaging in political discussion with friends, the timing of these discussions, as well as the interaction between political discussion and the level of prejudice among adolescents' peers.

We find that engaging in political discussion is significantly associated with lower levels of between-individual prejudice. However, within-subject fluctuation in the amount of discussion is only weakly related to levels of prejudice over time, although the relationship between political discussion and prejudice does increase with age. Results also demonstrate that the effect of political discussion with peers on prejudice depends on the level of prejudice among peers. We find that political discussions only matter for adolescent prejudice when peers hold relatively positive attitudes toward immigrants. Although we are unable to explain this relationship further, this result is consistent with other studies on prejudice.

There are limitations to our study. First, we only study Swedish adolescents from one city. While there is no theoretical reason to assume the relationships we find would be substantively different elsewhere, this remains an empirical question that our data do not permit us to explore. Moreover, these data do not identify the exact content of the political discussions, so we do not know the impact of discussing specific topics on attitudes. Relatedly, data collection occurred before the so-called “migration crisis” in 2015 and the related upsurge of immigration-specific content in media coverage and in political debates. While it is not unreasonable to assume that this priming would affect the content of political discussions, which would in turn influence how they relate to prejudicial attitudes, we lack the data to test this specifically. On the other hand, the timing of the study is also a strength in that this relationship is evident during less turbulent, and in this sense more representative, times. Finally, although the analyses rely on longitudinal data and we have controlled for a variety of key variables, including one's own political interest and other types of discussions with friends, because we do not have an experimental design, we cannot rule out omitted variable bias.

This brings us to the important question of causality and how we should understand our results. Three common criteria are associated with determining causality and causal order: (1) temporal precedence, in that x occurs prior to y; (2) covariation, in that x and y covary; and (3) the absence of other alternatives. We do meet the first and second criteria; however, we have not met the third. Despite the inclusion of a number of theoretical controls, we cannot be absolutely certain that we have controlled for all possible time-varying covariates. In fairness, this third criterion is arguably impossible to meet without an experimental research design. Nevertheless, we want to be responsible in our interpretation. Because we cannot rule out that some unmeasured factor matters for our results, do not claim causality outright. However, choosing to be conservative here does not mean that we cannot claim that we have shown associations with a temporal order. In this important regard, we improve upon previous cross-sectional studies that cannot.

As mentioned previously, future research should examine further why the impact of political discussion on prejudice does not appear important in high prejudice social networks. Another promising avenue for investigation is whether attitudes also guide adolescents' willingness to participate in political discussions. In a country such as Sweden, holding and expressing prejudicial attitudes is generally not socially acceptable. In line with the spiral of silence theory (Noelle-Neumann, 1974; Glynn et al., 1997), it is possible that strong norms against expressing negative attitudes toward out-groups may lead prejudiced adolescents to refrain from discussing politics with friends. Indeed, our results reveal a stronger between- rather than within-person effect of political discussion on prejudice, demonstrating that most of the variation is explained by differences between adolescents rather than within adolescents over time. We also find preliminary support for the inverse relationship: more prejudice means less political discussion. This suggests that, to the extent that these relationships are causal, they likely go in both directions. Thus, further research should seek to closer establish what is likely to be a complex interplay between the development of prejudice and engaging in political discussion over the course of adolescence. Future research should also examine why political discussions matter more as adolescents age. Although we review a number of plausible explanations based on previous research, our data do not permit us to adjudicate among these accounts. Another important task is to further examine the consequences of political discussion with significant others who are friends, including other classmates, teachers, and other adult role models. Finally, future studies should examine other types of social interactions beyond political discussion, as well as other forms of communication more generally.

Despite these shortcomings, this research makes several important contributions. First, we move beyond classic research in the field of prejudice that investigates *with whom* people interact by asking instead if *how* people interact matters for attitudes. Second, we show how political discussions is associated with prejudice over time by analyzing how it varies by age and by the level of prejudice in one's peer network. Third, in addition to the literature on prejudice, we contribute to a number of other areas of scholarship: political socialization, deliberative democracy, and research on attitude formation during the impressionable years.

## Ethics Statement

No ethics review is necessary for this article. The analysis of these data does not require that the authors handle nor contain sensitive personal data (e.g., race, political opinions, health, sexual preferences, and biometric data according to Swedish regulations) that can be connected to individuals. These data were collected by the Youth and Society Project at Örebro University, which was ethically reviewed and approved by the Regional Ethics Board in Uppsala.

## Author Contributions

The original idea is the result of group effort. All authors contributed to the development of the research question, hypotheses, and analytical strategy. All authors discussed and approved all analyses and content. AB ran the majority of the analyses and contributed to the writing of the text. MH contributed to writing the text. ME contributed to the writing the text.

### Conflict of Interest

The authors declare that the research was conducted in the absence of any commercial or financial relationships that could be construed as a potential conflict of interest.

## Footnotes

^1^While AIC slightly favors a second order autoregressive structure (9495.919 vs. 9492.361), we rely on BIC (9554.526 vs. 9557.481) which is more conservative in how it penalizes complex models. As a robustness check, we ran all models with a second order error structure and the results are the same. These results are available from the authors upon request.

^2^Cohort two did not participate at T4. For reasons of clarity we imputed the average of T3 and T5 at T4 for cohort 2 in Figures 1, 2. No such imputation is included in any of the analyses.

^3^Additional analyses suggest that fluctuations in prejudice also drive participation in political discussion. While testing this relationship more thoroughly requires different theoretical controls (consistent with explanations of and previous research on political discussions among youth), reversing the dependent and independent variable in model 1 reveals a negative between-effect (*b* = −0.22 SE = 0.02) as well as a negative within-effect (*b* = −0.05 SE = 0.02) of anti-immigrant attitudes on political discussions. This suggests that in addition to the negative effect of political discussions on prejudice, anti-immigrant sentiment may also make political discussion less likely.

^4^We have run additional analyses to test whether reciprocal nominations moderate the effect of political discussions, but there is no significant interaction effect.

^5^We have run the analysis separately in cohort 1 and cohort 2 to account for the possibility that cohort differences drive the relationship. These analyses reveal significant interaction effects in both cohort 1 (*b* = −0.04 SE = 0.02) and cohort 2 (*b* = −0.05 SE = 0.02) which provides further support for the interpretation that political discussions become more effective as adolescents age. We have also run the analysis without time to account for the risk of multi-collinearity.

## References

[B1] Aboud F. E. Doyle A. B. (1996). Does talk of race foster prejudice or tolerance in children? Can. J. Behav. Sci. 28, 161–170. 10.1037/0008-400X.28.3.161

[B2] Aboud F. E. Fenwick V. (1999). Exploring and evaluating school-based interventions to reduce prejudice. J. Soc. Issues 55, 767–785. 10.1111/0022-4537.00146

[B3] Abrams D. Hogg M. A. (1990). Social identification, self-categorization and social influence. Eur. Rev. Soc. Psychol. 1, 195–228. 10.1080/14792779108401862

[B4] Adelson J. O'Neil R. P. (1966). Growth of political ideas in adolescence: the sense of community. J. Pers. Soc. Psychol. 4, 295–306. 10.1037/h0023699 5969156

[B5] Alwin D. F. Krosnick J. A. (1991). Aging, cohorts, and the stability of sociopolitical orientations over the life span. Am. J. Sociol. 97, 169–195. 10.1086/229744

[B6] Amnå E. Ekström M. Kerr M. Stattin H. (2010). Du och Samhället (Youth and Society) Codebook (Vol. 2010). Örebro: Örebro University.

[B7] Asch S. E. (1956). Studies of independence and conformity: I. A minority of one against a unanimous majority. Psychol. Monogr. Gen. Appl. 70, 1–70. 10.1037/h0093718

[B8] Bandura A. (1977). Social Learning Theory. Englewood Cliffs, NJ: Prentice Hall.

[B9] Berndt T. J. (1979). Developmental changes in conformity to peers and parents. Dev. Psychol. 15, 608–616. 10.1037/0012-1649.15.6.608

[B10] Bessette J. (1980). Deliberative democracy: the majority principle in republican government, in How Democratic Is the Constitution? eds Goldwin R. Shambra W. (Washington, DC: American Enterprise Institute), 102–116.

[B11] Binder A. R. Dalrymple K. E. Brossard D. Scheufele D. A. (2009). The soul of a polarized democracy: testing theoretical linkages between talk and attitude extremity during the 2004 presidential election. Commun. Res. 36, 315–340. 10.1177/0093650209333023

[B12] Broockman D. Kalla J. (2016). Durably reducing transphobia: a field experiment on door-to-door canvassing. Science 352, 220–224. 10.1126/science.aad9713 27124458

[B13] Burnstein E. Vinokur A. D. (1977). Persuasive argumentation and social comparison as determinants of attitude polarization. J. Exp. Soc. Psychol. 13, 315–332. 10.1016/0022-1031(77)90002-6

[B14] Butrus N. Witenberg R. T. (2013). Some personality predictors of tolerance to human diversity: the roles of openness, agreeableness, and empathy. Aust. Psychol. 48, 290–298. 10.1111/j.1742-9544.2012.00081.x

[B15] Castillo J. C. Miranda D. Bonhomme M. Cox C. Bascopé M. (2015). Mitigating the political participation gap from the school: the roles of civic knowledge and classroom climate. J. Youth Stud. 18, 16–35. 10.1080/13676261.2014.933199

[B16] Coenders M. Lubbers M. Scheepers P. Verkuyten M. (2008). More than two decades of changing ethnic attitudes in the Netherlands. J. Soc. Issues 64, 269–285. 10.1111/j.1540-4560.2008.00561.x

[B17] Crandall C. S. Eshleman A. O'Brien L. (2002). Social norms and the expression and suppression of prejudice: the struggle for internalization. J. Pers. Soc. Psychol. 82, 359–378. 10.1037/0022-3514.82.3.359 11902622

[B18] Dejaeghere Y. Hooghe M. Claes E. (2012). Do ethnically diverse schools reduce ethnocentrism? A two-year panel study among majority group late adolescents in Belgian schools. Int. J. Intercult. Relat. 36, 108–117. 10.1016/j.ijintrel.2011.02.010

[B19] Deutsch M. Gerard H. B. (1955). A study of normative and informational social influences upon individual judgment. J. Abnorm. Soc. Psychol. 51, 629–636. 10.1037/h0046408 13286010

[B20] Dryzek J. S. (2000). Deliberative Democracy and Beyond: Liberals, Critics, Contestations. Oxford: Oxford University Press.

[B21] Eveland W. P. McLeod J. M. Horowitz E. M. (1998). Communication and age in childhood political socialization: an interactive model of political development. J. Mass Commun. Q. 75, 699–718. 10.1177/107769909807500406

[B22] Fearon J. D. (1998). Deliberation as discussion, in Deliberative Democracy, ed Elster J. (Cambridge: Cambridge University Press), 44–68.

[B23] Fishkin J. S. (2011). When the People Speak: Deliberative Democracy and Public Consultation. Oxford: Oxford University Press. Retrieved from: http://sfxeu11.hosted.exlibrisgroup.com/sfxumub?url_ver=Z39.88-2004&ctx_ver=Z39.88-2004&ctx_enc=info:ofi/enc:UTF-8&rfr_id=info:sid/sfxit.com:opac_856&url_ctx_fmt=info:ofi/fmt:kev:mtx:ctx&sfx.ignore_date_threshold=1&rft.object_id=3710000000376346&svc_val_fmt=info:ofi/fmt:kev:mtx:sch_svc&

[B24] Galinsky A. D. Moskowitz G. B. (2000). Perspective-taking: decreasing stereotype expression, stereotype accessibility, and in-group favoritism. J. Pers. Soc. Psychol. 78, 708–724. 10.1037/0022-3514.78.4.708 10794375

[B25] Glynn C. J. Hayes A. F. Shanahan J. (1997). Perceived support for one's opinions and willingness to speak out: a meta-analysis of survey studies on the “spiral of silence”. Pub. Opin. Q. 61, 452–463. 10.1086/297808

[B26] Gotlieb M. R. Kyoung K. Gabay I. Riddle K. Shah D. V. (2015). Socialization of lifestyle and conventional politics among early and late adolescents. J. Appl. Dev. Psychol. 41, 60–70. 10.1016/j.appdev.2015.06.004

[B27] Hatemi P. K. Funk C. L. Medland S. E. Maes H. M. Silberg J. L. Martin N. G.. (2009). Genetic and environmental transmission of political attitudes over a life time. J. Polit. 71, 1141–1156. 10.1017/S0022381609090938

[B28] Hjerm M. (2009). Anti-immigrant attitudes and cross-municipal variation in the proportion of immigrants. Acta Sociol. 52, 47–62. 10.1177/0001699308100633

[B29] Hjerm M. Eger M. A. Danell R. (2018). Peer attitudes and the development of prejudice in adolescence. Socius 4:2378023118763187. 10.1177/2378023118763187

[B30] Hogg M. A. Smith J. R. (2007). Attitudes in social context: a social identity perspective. Eur. Rev. Soc. Psychol. 18, 89–131. 10.1080/10463280701592070

[B31] Hooghe M. Boonen J. (2015). The intergenerational transmission of voting intentions in a multiparty setting: an analysis of voting intentions and political discussion among 15-year-old adolescents and their parents in Belgium. Youth Soc. 47, 125–147. 10.1177/0044118X13496826

[B32] Hooghe M. Meeusen C. Quintelier E. (2013) The impact of education intergroup friendship on the development of ethnocentrism. A latent growth curve model analysis of a five-year panel study among Belgian late adolescents. Eur. Soc. Rev. 29, 1109–1121. 10.1093/esr/jcs086

[B33] Isenberg D. J. (1986). Group polarization: a critical review and meta-analysis. J. Pers. Soc. Psychol. 50, 1141–1151. 10.1037/0022-3514.50.6.1141

[B34] Jennings M. K. Stoker L. Bowers J. (2009). Politics across generations: family transmission reexamined. J. Polit. 71, 782–799. 10.1017/S0022381609090719

[B35] Kaplan M. F. Miller C. E. (1987). Group decision making and normative versus informational influence: effects of type of issue and assigned decision rule. J. Pers. Soc. Psychol. 53, 306–313. 10.1037/0022-3514.53.2.306

[B36] Keijsers L. Frijns T. Branje S. J. T. Meeus W. (2009). Developmental links of adolescent disclosure, parental solicitation, and control with delinquency: moderation by parental support. Dev. Psychol. 45, 1314–1327. 10.1037/a0016693 19702394

[B37] Kiesner J. Maass A. Cadinu M. Vallese I. (2003). Risk factors for ethnic prejudice during early adolescence. Soc. Dev. 12, 288–308. 10.1111/1467-9507.00234

[B38] Krosnick J. A. Alwin D. F. (1989). Aging and susceptibility to attitude change. J. Pers. Soc. Psychol. 57, 416–425. 10.1037/0022-3514.57.3.416 2778632

[B39] Larson R. W. Richards M. H. Moneta G. Holmbeck G. Duckett E. (1996). Changes in adolescents' daily interactions with their familieis from ages 10-18: disengagement and transformation. Dev. Psychol. 32, 744–754. 10.1037/0012-1649.32.4.744

[B40] Luna B. Garver K. E. Urban T. A. Lazar N. A. Sweeney J. A. (2004). Maturation of cognitive processes from late childhood to adulthood. Child Dev. 75, 1357–1372. 10.1111/j.1467-8624.2004.00745.x 15369519

[B41] McDevitt M. (2006). The partisan child: developmental provocation as a model of political socialization. Int. J. Pub. Opin. Res. 18, 67–88. 10.1093/ijpor/edh079

[B42] McLeod J. M. (2000). Media and civic socialization of youth. J. Adolesc. Health 27, 45–51. 10.1016/S1054-139X(00)00131-2 10904206

[B43] McLeod J. M. Shah D. V. (2009). Communication and political socialization: challenges and opportunities for research. Polit. Commun. 26, 1–10. 10.1080/10584600802686105

[B44] Meeusen C. (2014). The intergenerational transmission of environmental concern: the influence of parents and communication patterns within the family. J. Environ. Educ. 45, 77–90. 10.1080/00958964.2013.846290

[B45] Meeusen C. Dhont K. (2015). Parent–child similarity in common and specific components of prejudice: the role of ideological attitudes and political discussion. Eur. J. Pers. 29, 585–598. 10.1002/per.2011

[B46] Miklikowska M. (2018). Empathy trumps prejudice: the longitudinal relation between empathy and anti-immigrant attitudes in adolescence. Dev. Psychol. 54, 703–717. 10.1037/dev0000474 29239638

[B47] Miklikowska M. Bohman A. Titzmann P. F. (2019). Driven by context? The interrelated effects of parents, peers, classrooms on development of prejudice among Swedish majority adolescents. Dev. Psychol. 10.1037/dev0000809 31512893

[B48] Mitchell J. (2019). Prejudice in the classroom: a longitudinal analysis of anti-immigrant attitudes. Ethn. Racial Stud. 42, 1514–1533. 10.1080/01419870.2018.1493209

[B49] Moscovici S. Zavalloni M. (1969). The group as a polarizer of attitudes. J. Pers. Soc. Psychol. 12, 125–135. 10.1037/h0027568

[B50] Mutz D. C. Mondak J. J. (2006). The workplace as a context for cross-cutting political discourse. J. Polit. 68, 140–155. 10.1111/j.1468-2508.2006.00376.x

[B51] Myers D. G. Bishop G. D. (1970). Discussion effects on racial attitudes. Science 169, 778–779. 10.1126/science.169.3947.778 4914703

[B52] Myers D. G. Lamm H. (1976). The group polarization phenomenon. Psychol. Bull. 83, 602–627. 10.1037/0033-2909.83.4.602

[B53] Nesdale D. Griffith J. Durkin K. Maass A. (2005). Empathy, group norms and children's ethnic attitudes. J. Appl. Dev. Psychol. 26, 623–637. 10.1016/j.appdev.2005.08.003

[B54] Niemi R. G. Jennings M. K. (1991). Issues and inheritance in the formation of party identification. Am. J. Pol. Sci. 35, 970–988. 10.2307/2111502

[B55] Noelle-Neumann E. (1974). The spiral of silence a theory of public opinion. J. Commun. 24, 43–51. 10.1111/j.1460-2466.1974.tb00367.x

[B56] Orey B. D. A. Park H. (2012). Nature, nurture, and ethnocentrism in the Minnesota twin study. Twin Res. Hum. Genet. 15, 71–73. 10.1375/twin.15.1.71 22784455

[B57] Paluck E. L. (2011). Peer pressure against prejudice: a high school field experiment examining social network change. J. Exp. Soc. Psychol. 47, 350–358. 10.1016/j.jesp.2010.11.017

[B58] Pattie C. J. Johnston R. J. (2008). It's good to talk: talk, disagreement and tolerance. Br. J. Polit. Sci. 38, 677–698. 10.1017/S0007123408000331

[B59] Pettigrew T. F. (1998). Intergroup contact theory. Annu. Rev. Psychol. 49, 65–85. 10.1146/annurev.psych.49.1.65 15012467

[B60] Pettigrew T. F. Tropp L. R. (2011). When Groups Meet: The Dynamics of Intergroup Contact. New York, NY: Psychology Press.

[B61] Poteat V. P. (2007). Peer group socialization of homophobic attitudes and behavior during adolescence. Child Dev. 78, 1830–1842. 10.1111/j.1467-8624.2007.01101.x 17988324

[B62] Poteat V. P. Espelage D. L. Green H. D. Jr. (2007). The socialization of dominance: peer group contextual effects on homophobic and dominance attitudes. J. Pers. Soc. Psychol. 92, 1040–1050. 10.1037/0022-3514.92.6.1040 17547487

[B63] Price V. Cappella J. N. Nir L. (2002). Does disagreement contribute to more deliberative opinion? Polit. Commun. 19, 95–112. 10.1080/105846002317246506

[B64] Price V. Nir L. Cappella J. N. (2006). Normative and informational influences in online political discussions. Commun. Theory 16, 47–74. 10.1111/j.1468-2885.2006.00005.x

[B65] Quintelier E. (2015). Engaging adolescents in politics. Youth Soc. 47, 51–69. 10.1177/0044118X13507295

[B66] Raabe T. Beelmann A. (2011). Development of ethnic, racial, and national prejudice in childhood and adolescence: a multinational meta-analysis of age differences. Child Dev. 82, 1715–1737. 10.1111/j.1467-8624.2011.01668.x 22023224

[B67] Rekker R. Keijsers L. Branje S. Meeus W. (2015). Political attitudes in adolescence and emerging adulthood: developmental changes in mean level, polarization, rank-order stability, and correlates. J. Adolesc. 41, 136–147. 10.1016/j.adolescence.2015.03.011 25880889

[B68] Schneider S. L. (2008). Anti-immigrant attitudes in Europe: outgroup size and perceived ethnic threat. Eur. Sociol. Rev. 24, 53–67. 10.1093/esr/jcm034

[B69] Sears D. O. Valentino N. A. (1997). Politics matters: political events as catalysts for preadult socialization. Am. Polit. Sci. Rev. 91, 45–65. 10.2307/2952258

[B70] Sherif M. Sherif C. W. (1953). Groups in Harmony and Tension; an Integration of Studies of Intergroup Relations. Oxford: Harper & Brothers.

[B71] Sinclair S. Dunn E. Lowery B. (2005). The relationship between parental racial attitudes and children's implicit prejudice. J. Exp. Soc. Psychol. 41, 283–289. 10.1016/j.jesp.2004.06.003

[B72] Stearns E. Buchmann C. Bonneau K. (2009). Interracial friendships in the transition to college: do birds of a feather flock together once they leave the nest? Sociol. Educ. 82, 173–195. 10.1177/003804070908200204

[B73] Tajfel H. Turner J. C. (1979). An intergrative theory of intergroup conflict, in The Social Psychology of Intergroup Relations, eds Austin W. G. Worchel S. (Monterey, CA: Brooks/Cole), 33–47.

[B74] Thijs J. Gharaei N. de Vroome T. (2016). Why should I?”: adolescents' motivations to regulate prejudice in relation to their norm perceptions and ethnic attitudes. Int. J. Intercult. Relat. 53, 83–94. 10.1016/j.ijintrel.2016.05.006

[B75] Turner J. C. (1981). The experimental social pshycology of intergroup behaviour, in Intergroup Behaviour, eds Turner J. C. Giles H. (Oxford: Blackwell, 66–101.

[B76] Turner J. C. (1991). Social Influence. Milton Keynes; Pacific Grove: Open University Press; Brooks/Cole.

