# Supplementary material for: Politics and Prejudice: How Political Discussion With Peers Is Related to Attitudes About Immigrants During Adolescence
Source: Front Sociol. 2019 Oct 4;4:70. doi: 10.3389/fsoc.2019.00070 (PMC8022588; doi:10.3389/fsoc.2019.00070)
Supplement: Supplementary file 1 [file Table_1.pdf]

Table A1. Variables in the analysis (uncentered)

Variable	Description	Mean T1-T5	SD T1-T5	Min-Max
Dependent variable				
Prejudice	*See Data & Method*	2.27	.74	1-4
Independent variable				
Political discussions with friends	*See Data & Method*	2.21	.71	1-4
Moderators and main controls				
Age	*See Data & Method*	16.4	2.00	13-22
Friends' prejudice	*See Data & Method*	2.25	.47	1-4
Political interest	*See Data & Method*	3.05	.99	1-5
Additional controls				
Male	*0=female, 1=male*	.48	.50	0-1
Popularity	*Number of times respondent has been nominated by other*	4.70	2.59	0-15
Reciprocity	*Number of reciprocated nominations*	3.31	1.88	0-8
Feeling of loneliness	*I feel alone and like a stranger in my class. 1=Absolutely agree, 4=Absolutely disagree*	3.61	.65	1-4
Other discussions friends	*Index 6 variables: How often does it happen that you and your friends talk about (3) Films, music, and TV series, (4) Weekend activities, (5) School and school work, (6) Environmental issues, (7) Social media and Internet and (8) Computer gaming? 1=Never, 4=Very often*	2.96	.39	1-4
Political discussion parents	*Index 2 variables: How often does it happen that you and your parents talk about (1) What you have heard on the news about what is going on in Sweden and around the world (2) Politics or societal issues. 1=Never, 4=Very often*	2.44	.71	1-4
Parents prejudice	*Questions to parents. Index 3 variables: (1) It happens only too often that immigrants have customs and traditions that do not fit in into Swedish society. (2) Immigrants often come here just to take advantage of the welfare in Sweden. (3) Immigrants often take jobs from people who are born in Sweden 1=Doesn't apply at all, 4=Applies very well.*	2.04	.58	1-4
Parents education (mother/father)	*0= Less than University degree, 1=University degree*	.57/.38	.50/.49	0-1

Table A2. Political discussions and prejudice, additional controls

	Model A1	Model A2	Model A3
Fixed			
Intercept	2.47(.10)***	2.02(.18)***	1.31 (.24)***
T1 (ref)			
T2	.08(.06)	.11(.06)	.14(.07)*
T3	.06(.06)	.08(.06)	.08(.07)
T4	.08(.07)	.10 (.07)	.10 (.08)
T5	-.11(.07)	-.10(.07)	-.08 (.08)
Political discussion (w)	-.05(.02)**	-.03(.02)	-.03 (.02)
Political discussion (b)	-.15(.04)***	-.17(.04)***	-.22 (.05)***
Controls			
Friends' prejudice (w)	.20(.03)***	.21(.03)***	.19 (.03)***
Friends' prejudice (b)	.57(.04)***	.57(.04)***	.50(.05)***
Political interest (w)	-.02(.01)	-.02(.01)*	-.02 (.01)*
Political interest (b)	-.06(.02)*	-.07(.03)**	-.05 (.03)
Age	.03(.01)**	.03(.01)***	.04(.01)**
Male	.13(.03)***	.11(.03)***	.17(.04)***
Popularity (w)	-.00(.01)		-.01 (.01)
Popularity (b)	.01(.01)		.01 (.02)
Reciprocity (w)	.00(.01)		.01 (.01)
Reciprocity (b)	-.02(.02)		-.02(.02)
Feeling of loneliness (w)	.02(.02)		.01(.02)
Feeling of loneliness (b)	-.11(.03)***		-.10 (.04)**
Other discussions friends (w)		-.05(.03)	-.01(.04)
Other discussions friends (b)		.06(.05)	.12 (.07)
Political discussion parents (w)		-.03(.02)	-.03(.02)
Political discussion parents (b)		-.01(.04)	.06(.04)
Parents' prejudice			.28 (.03)***
Father's education			-.11(.04)**
Random			
Time	.04(.03)	.05(.02)	.04(.03)
Intercept	.32(.03)	.33(.03)	.28(.04)
Residual (Ar1)			
Rho	.40(.04)	.39(.04)	.40(.05)
Sd(e)	.58(.02)	.57(.02)	.57(.02)
n	1433	1440	961
Obs	4212	4363	2981
BIC	7771.063	8028.638	5395.657
AIC	7625.112	7894.639	5221.657

Note: Standard errors in brackets.*p < .05.**p < .01.***p < .001. (w)=within-person effects, (b)=between-person effects
